# Mass Spectrometry-Based Quantification of the Antigens in Aluminum Hydroxide-Adjuvanted Diphtheria-Tetanus-Acellular-Pertussis Combination Vaccines

**DOI:** 10.3390/vaccines10071078

**Published:** 2022-07-05

**Authors:** Larissa van der Maas, Maarten Danial, Gideon F. A. Kersten, Bernard Metz, Hugo D. Meiring

**Affiliations:** 1 Intravacc, Antonie van Leeuwenhoeklaan 9, 3721 MA Bilthoven, The Netherlands; maarten.danial@intravacc.nl (M.D.); g.f.a.kersten@lacdr.leidenuniv.nl (G.F.A.K.); bernard.metz@intravacc.nl (B.M.); hugo.meiring@intravacc.nl (H.D.M.); 2 Division of BioTherapeutics, Leiden Academic Centre for Drug Research (LACDR), Leiden University, 2333 CC Leiden, The Netherlands

**Keywords:** mass spectrometry, vaccines, proteomics, 3Rs

## Abstract

Vaccines undergo stringent batch-release testing, most often including in-vivo assays for potency. For combination vaccines, such as diphtheria-tetanus-pertussis (DTaP), chemical modification induced by formaldehyde inactivation, as well as adsorption to aluminum-based adjuvants, complicates antigen-specific in-vitro analysis. Here, a mass spectrometric method was developed that allows the identification and quantitation of DTaP antigens in a combination vaccine. Isotopically labeled, antigen-specific internal standard peptides were employed that permitted absolute quantitation of their antigen-derived peptide counterparts and, consequently, the individual antigens. We evaluated the applicability of the method on monovalent non-adjuvanted antigens, on final vaccine lots and on experimental vaccine batches, where certain antigens were omitted from the drug product. Apart from the applicability for final batch release, we demonstrated the suitability of the approach for in-process control monitoring. The peptide quantification method facilitates antigen-specific identification and quantification of combination vaccines in a single assay. This may contribute, as part of the consistency approach, to a reduction in the number of animal tests required for vaccine-batch release.

## 1. Introduction

Animal tests are commonly performed for the batch-release testing of many vaccines. However, vaccine manufacturers and regulatory authorities are looking for new methods to replace release testing in animals by in-vitro assays. The so-called consistency approach has been identified as an option to reduce, replace or refine the number of animals for quality control. This approach is based on consistent production of specific vaccine products, which can be demonstrated by lot-to-lot testing, showing comparable characteristics and properties [1]. Batch consistency is demonstrated with a panel of in-vitro assays that assess physicochemical, biological, immunochemical and purity aspects of the vaccine. From the information gained, this strategy may lead to a substantial reduction in animal use [2]. For example, DTaP vaccines, containing diphtheria toxoid (DTd), tetanus toxoid (TTd) and pertussis antigens, undergo strict lot-release testing on animals. The potency of these vaccines is assessed based on immunogenicity and, in some cases, challenge models performed on guinea pigs or mice [3,4,5].

The content of individual antigens in the final drug product is controlled during the formulation process. Before specific amounts of individual drug substances are combined, the antigen concentrations of the drug substances are determined by the Kjeldahl method or via immunoassays (e.g., ELISA or flocculation tests). After blending, determination of specific antigen content is challenging due to the presence of multiple antigens as well as aluminum adjuvants in the drug product matrix. Nevertheless, several assays have been developed to quantify antigens in aluminum-adjuvanted vaccines. For example, the o-phthalaldehyde (OPA) fluorescent protein assay can directly and accurately determine total protein content in vaccine formulations [6]. Alternatively, a direct Alhydrogel formulation immunoassay (DAFIA) can be applied to determine antigen content, identity and the integrity of formulations containing aluminum hydroxide [7]. Further, an enzyme-linked immunosorbent assay (ELISA) and DAFIA tests have been developed to specifically quantify DTd in final vaccine lots [8,9]. Flowcytometry has also been demonstrated to enable specific antigen quantitation for individual recombinant protein antigens in an aluminum-adjuvanted Neisseria meningitidis serogroup B combination vaccine [10]. However, no similar assays have been developed for DTaP vaccines. Furthermore, the assays that have been developed do not permit simultaneous identification and specific quantitation of antigens in final combination vaccines. To this end, we focused on liquid chromatography–mass spectrometry (LC-MS) in tackling these challenges.

LC-MS is a state-of-the-art tool to characterize complex protein samples, such as combination vaccines. Targeted mass spectrometry is used as a method to quantify proteins of interest with high sensitivity, accuracy and reproducibility [11]. This method is widely applied in vaccine research, for example, for the quantification of neuramidase and hemagglutinin in influenza vaccine [12], as well as the detection of residual UL5 and UL29 proteins in a human herpes simplex virus (HSV)-2 viral vaccine candidate [13]. In addition, quantitative MS-based assays were developed to (i) quantify the amount of PorA and PorB in Neisseria meningitidis outer membrane vesicles (OMVs) adsorbed to aluminum hydroxide [14,15] and (ii) identify and quantify the Bordetella pertussis antigens: Pertussis toxoid (PTd), filamentous hemagglutinin (FHA), pertactin (PRN) and fimbriae 2 (Fim2) in DTaP vaccines [16]. However, in the latter work, the label-free LC-MS method developed for the pertussis antigens was not used to quantify the DTd and TTd components of the DTaP vaccines, and it was unclear from this study whether the vaccine samples were adsorbed to aluminum adjuvants.

Currently, there is no single assay available to quantify all antigens in DTaP vaccines following adsorption to aluminum adjuvants. Therefore, we developed a targeted LC-MS [17] method that allows the identification and quantitation of all antigens in DTaP vaccines within a single analytical workflow using antigen-specific synthetic peptides corresponding to target antigen sequences as internal standards. These standards must be individually selected and screened for suitability for each antigen present in the vaccine analyte. Bacterial toxins used in vaccines are generally inactivated with formaldehyde, resulting in chemical modifications in the proteins. Research by Metz et al. [18] demonstrated that formaldehyde treatment predominantly results in the chemical modification of arginine, lysine and tyrosine and, to a lesser extent, of asparagine, glutamate, histidine and tryptophan, preventing the use of trypsin in this method [19]. By contrast, the metalloprotease Asp-N is a very promising protease for this application, since the proteolysis may be less affected by formaldehyde modifications [20,21]. Within this research, we developed a mass-spectrometry-based method for the quantification of the antigens in a final DTaP vaccine.

## 2. Materials and Methods

**Antigens.** DTd, TTd, FHA and PTd were supplied by an industrial partner of the Vac2Vac consortium and anonymized in accordance with agreements made within this consortium. The manufacturer that provided the antigens and the DTaP vaccines will from this point forward be named as ‘manufacturer’. All samples were stored at 4 °C upon arrival.

**Stable isotopically labeled internal standard peptides.** Stable isotopically labeled antigen-specific internal standard peptides were purchased from Pepscan (Lelystad, The Netherlands), each containing a single amino acid labeled with ^13^C and ^15^N in lieu of ^12^C and ^14^N, respectively.

**Vaccines.** Two batches of an adjuvanted, multivalent DTaP vaccine were provided by the manufacturer (part of the Vac2Vac consortium). In addition, batches of control vaccines were provided, for each of which, one antigen was omitted.

**Protein determination by UV absorption.** Protein content of individual non-adsorbed antigens was determined in duplicate by 280 nm UV absorbance [22] using a plate reader equipped with Take3 plates (Synergy Neo 2, BioTek, Winooski, VT, USA) and 3 µL of sample. The background signal was determined in duplicate with ultrapure water.

**SDS-PAGE.** Digested (1 µg) and undigested (1 µg) antigens were mixed with 4× reducing sample buffer (250 mM Tris; Merck), 8% (*w*/*v*) SDS (Sigma Aldrich, Saint Louis, MO, USA), 400 mM DTT (Sigma Aldrich), 40% (*v*/*v*) glycerol (Sigma Aldrich) and 0.04% (*w*/*v*) bromophenol blue (Sigma Aldrich) and incubated for 10 min at 100 °C. Samples were loaded onto 10% (*w*/*v*) NuPAGE Bis-Tris 1.0 mm precast gels (Thermo Fisher, Waltham, MA, USA) and proteins were separated in MES running buffer (Thermo Fisher), at 200 Volt (V) for 45 min in an XCell SureLock minicell system (Thermo Fisher). The gel was stained for 1 h with Coomassie (Imperial Protein Stain; Thermo Fisher), destained for 24 h with ultrapure water and imaged using the Octoplus QPLEX imager (NH DyeAGNOSTICS, Halle, Germany).

**Enzymatic digestion of antigens by Asp-N.** Individual antigens were dialyzed against 50 mM phosphate buffer pH 7.4 (1 M stock, Sigma Aldrich) using a 3 kDa MWCO membrane (Sigma Aldrich), after which a protein determination by UV absorption was performed as described above. Proteins were denatured in 0.1% RapiGest (Waters, Milford, MA, USA) for 30 min incubation at 80 °C. Antigen (2 µg) was diluted in a total volume of 15 µL with ultrapure water. Phosphate buffer (15 µL of 100 mM, pH 7.4) was added to the antigen. All antigens were reduced and alkylated before digestion. This was done by adding Tris(2-carboxyethyl)phosphine hydrochloride (TCEP) (Thermo Scientific, Waltham, MA, USA) (1 µL, 200 mM in ultrapure water) followed by an incubation at 55 °C for 1 h. This was followed by 30 min incubation in the dark with 1 µL 375 mM iodoacetamide (Thermo Fisher) dissolved in 100 mM phosphate buffer pH 7.4. Antigens were digested overnight at 37 °C with Asp-N (Roche, Basel, Switzerland) using three different enzyme:antigen (*w*/*w*) ratios: 1:20 (100 ng enzyme), 1:50 (40 ng enzyme) and 1:100 (20 ng enzyme). Trifluoroacetic acid (TFA) (Biosolve) was added to the samples to a final concentration of 1% and incubated for 1 h at RT. Samples were centrifugated for 15 min after which 5 µL of the clear supernatant was diluted to 50 µL with ultrapure water containing 0.1% formic acid (FA) (Merck, Darmstadt, Germany), and 5% DMSO (*v*/*v*/*v*) (Sigma Aldrich), from now on named ‘diluent’.

**Quantification of DTaP antigens in final vaccine product.** The antigens present in 100 µL DTaP vaccine were denatured by adding RapiGest to a final concentration of 0.1% and incubation for 60 min at 80 °C. Next, 100 pmol of each internal standard peptide was added to the denatured vaccine, followed by reduction (5 µL 200 mM TCEP) and alkylation (5 µL 375 mM iodoacetamide). Digestion was conducted using 0.1 µg Asp-N incubated at 37 °C overnight. The samples were centrifugated for 5 min at 5000× *g*, 100 µL supernatant was collected and FA was added to the supernatant to a final concentration of 1% and incubated for 1 h at RT. The sample was cleaned up using automated solid phase extraction (SPE) (GX-271 ASPEC, Gilson, The Hague, The Netherlands) utilizing C18 columns (Waters). After the extraction, the samples were dried under reduced pressure (Concentrator Plus; Eppendorf, Hamburg, Germany) and reconstituted in 100 µL of diluent and analyzed using LC-MS.

**LC-MS analysis.** Digested peptide samples were analyzed by nanoscale reversed-phase liquid chromatography electrospray mass spectrometry [23]. Chromatographic separation was performed on an Agilent technologies 1290 infinity LC system. Peptides were loaded on a trapping column (Reprosil-Pur C18-AQ 5 µm (Dr. Maisch, Ammerbuch-Entringen, Germany); 20 mm long × 100 µm inner diameter, packed in house) using solvent A (0.1% (*v*/*v*) FA (Merck) in water) for 10 min at a column flow rate of 5 µL/min. The peptides were separated by reversed-phase chromatography on an analytical column (Reprosil-Pur C18-AQ 3 µm (Dr. Maish); 35 cm long × 50 µm inner diameter, packed in house) at a column flow rate of 125 nL/min. The gradient was started with 7.5% solvent B (0.1% (*v*/*v*) formic acid in acetonitrile (Biosolve, Valkenswaard, The Netherlands) to 57.5% in 25 min, followed by a step to 85% (hold for 10 min). After the gradient, the columns were equilibrated for 10 min in 100% solvent A at 125 nL/min to prepare for the next injection. The peptides were measured using an Orbitrap Fusion Lumos mass spectrometer (Thermo Scientific) by data-dependent scanning, comprising an MS-scan (*m*/*z* 350−1500) in the orbitrap with a resolution of 120,000 (FWHM), followed by collision-induced dissociation (CID) of the 10 most abundant ions (charge states between 1 and 5) of the MS spectrum and an Ion Trap readout. The threshold value for these precursor ions was set at 1000 counts. The normalized collision energy was set to 35% and isolation width at 1.6 Da, activation Q to 0.250 and activation time to 30 ms. The maximum injection time for MS scans was set to 50 ms and for signature peptide selection, MS/MS scans were set to 100 ms. Precursor ions with unknown charge states were excluded for MS/MS analysis. Dynamic exclusion was enabled with repeat set to 2 (if occurred within 15 s) and an exclusion duration of 30 s.

**Method for antigen quantification.** The antigen concentrations were calculated based on the chromatographic peak area ratio of the signature peptides: $n_{Ag}=\frac{\left(n_{SP}\times A_{Ag}\right)}{A_{SP}}$ where *n_Ag_* is the amount of the antigen (in pmol), *n_SP_* is the amount of isotopically labeled signature peptide (in pmol), and *A_Ag_* and *A_SP_* are the chromatographic peak areas for the monoisotopic ion traces of the unlabeled and the isotopically labeled signature peptides, respectively.

## 3. Results

### 3.1. Method Development Strategy

Quantification was assessed using mass spectrometry to determine the amount of DTd, TTd, FHA and PTd antigens in aluminumhydroxide-adsorbed DTaP vaccines. The experimental design comprised several steps: (i) selection of signature peptides, (ii) quantification of individual antigens and (iii) quantification of antigens in a DTaP vaccine batch from a single manufacturer.

### 3.2. Selection of Signature Peptides

In-silico digestion with Asp-N revealed 5 potential signature peptides for DTd, 35 for TTd, 62 for FHA and 5 for PTd, fulfilling the selection criteria for internal standard peptides. In brief, the signature peptides cannot contain cysteine (C), methionine (M) or tryptophan (W), because these amino acids are involved in the formation of disulfide bridges or are otherwise sensitive to oxidation [17]. Ideally, the signature peptide will have a high peak intensity, minimal enzymatic miscleavage (no peptide length variants) and a peptide length between 5 and 20 amino acids.

Actual digestion of the individual antigens and subsequent LC-MS analysis showed that not all in-silico proteolytic peptides were identified or abundantly recovered in the digest (Table S1). A positive selection of signature peptides was made based on their abundances in the LC-MS analysis.

### 3.3. Antigen Proteolysis

All antigens demonstrated at least partial proteolysis after treatment with the metalloprotease Asp-N. Asp-N was added in different enzyme:substrate weight ratios (1:20, 1:50 and 1:100). The digests were analyzed on SDS-PAGE (Figure S1). DTd and TTd exhibited complete proteolysis (Figure S1A,B). The digestion of FHA was not complete; however, the gel demonstrated that the band for intact FHA was completely absent and shifted to bands with lower molecular weight (Figure S1C). PTd did not show any subunit bands in the control lane, due to low-protein concentrations in the sample. The absence of these bands did not permit any further analysis by SDS-PAGE as to whether the PTd was sufficiently digested (Figure S1D).

**Figure 1:**
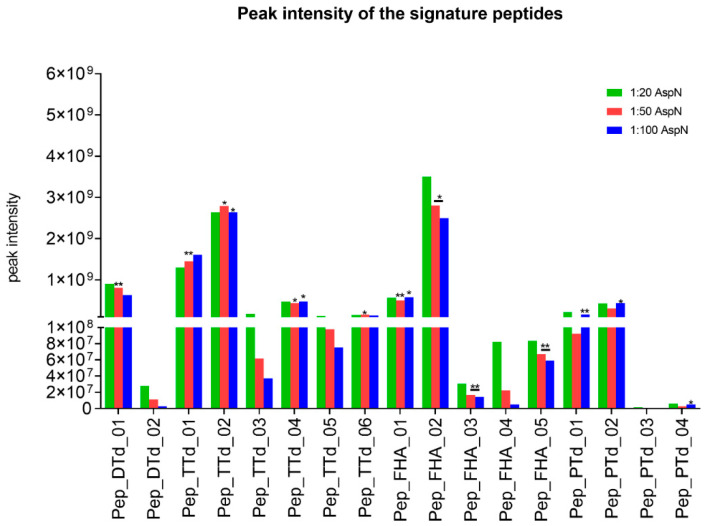
Peak intensity of the signature peptides as a function of the enzyme-to-substrate ratio. DTd, FHA, PTd and TTd were digested with three different Asp-N:antigen ratios (*w*/*w*): 1:20 (green bars), 1:50 (red bars) and 1:100 (blue bars). The different signature peptides are plotted on the *X*-axis and their peak heights are plotted on the *Y*-axis. Peak heights were compared to each other. * Peak height difference ≤ 10%, ** peak height difference ≤ 20%.

The chromatographic peak areas of the potential signature peptides were monitored for the three different enzyme:antigen ratios (1:20, 1:50 and 1:100, *w*/*w*) and, obviously, should be independent of the amount of enzyme used for digestion. Therefore, as a selection criterion, two out of three enzyme:antigen ratios should result in chromatographic peak areas with a maximum difference of 20%, to indicate optimal completeness of the digestion. The intensities for all potential target signature peptides were plotted, as shown in Figure 1.

For DTd, the response of one candidate signature peptide (pep_DTd_01) was independent of the enzyme-to-substrate ratio. Within the TTd peptides, this was the case for four selected signature peptides, but pep_TTd_01 and pep_TTd_02 showed the highest responses. Five candidate peptides were assessed for FHA. Pep_FHA_01 and pep_FHA_02 were chosen for antigen quantification because of the highest and most stable chromatographic peak areas in relation to different enzyme-to-substrate ratios. For quantification of PTd, pep_PTd_01 and pep_PTd_02 were detectable and suitable as signature peptides.

To this end, one or two target peptides were assigned to each antigen as potentially suitable signature peptides (Table 1). The stable isotopically labeled signature peptides were extended with 4–6 amino acids on the N-terminus or C-terminus matching the antigen sequence in order to create an Asp-N digestion site to allow for monitoring of digestion efficiency. Some internal standard peptides contain an amino acid, which potentially can be modified by formaldehyde or be deamidated. Digests were screened for the presence of such modifications. This analysis revealed that, at most, 2% of the signature peptides contained modified residues, which was considered acceptable. Therefore, all selected signature peptides (Table 1) were used for antigen quantification.

### 3.4. Linearity of the Response Ratio between Signature Peptides and Antigen Concentration

**Figure 2:**
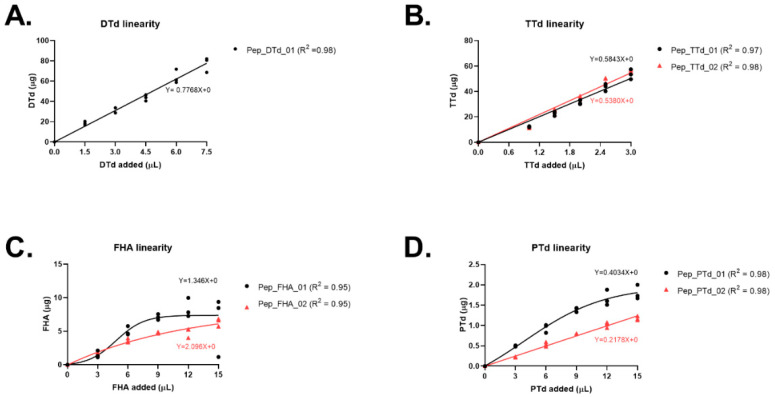
Linearity of the antigen concentrations of DTd (**A**), TTd (**B**), FHA (**C**) and PTd (**D**) using antigen-specific signature peptides. Different amounts of individual antigens were spiked with antigen specific internal standard peptide and digested using AspN. The amount of antigen is plotted versus the amount of antigen added in µL. Linear regression was plotted for each signature peptide.

Each individual antigen, in five different amounts and spiked with a fixed amount of its respective isotopically labeled internal standard peptide(s), was digested using AspN and their concentrations determined by mass spectrometry. For all antigens, the linear range was determined (R^2^ > 0.95) (Figure 2).

The DTd curve demonstrated good linearity (ranging from 0 to 80 µg) (Figure 2A). For TTd, two internal standard peptides (pep_TTd_01 and pep_TTd_02) were used, resulting in equal TTd amounts (ranging from 0 to 60 µg) (Figure 2B).

The amounts of FHA determined, however, were different for the two signature peptides and were not linear over the entire range tested. The amount of FHA using pep_FHA_01 (linear from 0 to6 µg) was two-fold higher compared to the amount of FHA using pep_FHA_02 (linear from 0 to 4.5 µg) (Figure 2C), this could be due to the structure of FHA and the lack of digestion efficiency in relation to the rigid rod structure of FHA [24].

As shown in Figure 2D, PTd could be quantified using two internal standard peptides. Pep_PTd_02 demonstrated a linear antigen amount (ranging from 0 to 0.75 µg). In contrast, quantification using pep_PTd_01 revealed comparable PTd quantities, but the concentration that could be determined appeared to reach a maximum at higher PTd volumes assayed and deviated from a linear response beyond the range of 0–1.4 µg (Figure 2D).

In summary, based on the linear curves generated for all the antigens, we conclude that the antigens DTd, PTd and TTd can be quantitated using mass spectrometry with the selected signature peptides as quantitation standards. For FHA, the apparent antigen concentration was different depending on which standard peptide was used. Without a clear cause of the discrepancy between the two FHA peptides, it was not possible to determine which was more accurate.

### 3.5. Quantification of the Antigens in Aluminum-Hydroxide-Adjuvanted Vaccine

Quantitation of antigens in final vaccine lots was performed using an aluminum-hydroxide-adjuvanted DTaP-Hib combination vaccine (Table 2).

**Figure 3:**
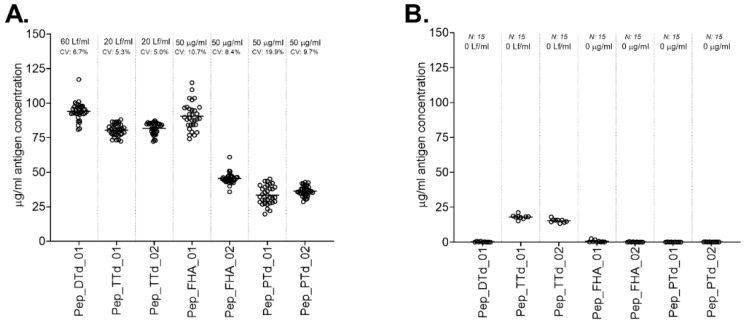
Antigen concentrations present in the DTaP vaccine and control vaccine (**A**) and the concentration of the missing antigen in its corresponding control DTaP vaccine (**B**). The nominal antigen concentrations and the CV of the analysis are depicted at the top of the graph. The used signature peptides are shown on the X-axis. To determine antigen concentration in the DTaP vaccine, 100 pmol of each internal standard peptide was added to 100 µL vaccine and digested with 0.33 µg Asp-N. (**A**): vaccine A (*n* = 36 for each antigen and signature peptide combination, except for pep_FHA_01 (*n* = 31) and pep_PTd_01 (*n* = 33)); (**B**): experimental (control) vaccines in which the quantified antigen was missing (*n* = 9 per signature peptide).

In addition, several negative control vaccine formulations were included in the study, in each of which, one of the antigens was omitted. This allowed us to evaluate the specificity of the method. Analysis of the control vaccines showed that the method was highly antigen specific (Figure 3A,B).

For TTd, PTd and FHA, the average protein concentration using both internal standard peptides is depicted in Table 3. In the control samples (Figure 3B), the antigen recoveries for all omitted antigens in their corresponding control formulations were zero or close to zero, except for TTd (pep_TTd_01 and pep_TTd_02). For the *Haemophilus influenzae* type b (Hib) conjugate vaccine, TTd is used as carrier for the polyribosyl ribitol phosphate polysaccharide [25]. Therefore, the control vaccine sample contains a detectable amount of TTd as the carrier. This assay was not optimized to specifically determine the concentration of the TTd carrier protein in such formulations.

For assessment of batch-to-batch comparison, the concentration of antigens was determined in a second batch (batch 2) of the DTaP vaccine. Table 3 and Figure S2 show the concentration of both batches.

A *t*-test was used to compare batch 1 and batch 2, whereby a *p*-value < 0.05 was considered significantly different. For DTd and TTd, this resulted in significantly different amounts of antigen quantified in batches 1 and 2 of the DTaP vaccine. While the cause of the differences in concentration observed between the two batches cannot be definitively assessed based on these experiments, it should be noted that the DTd and TTd antigens are formulated based on flocculation units (Lf/mL), which is the output of an immunological assay using DT- or TT-specific antisera. By contrast, the pertussis antigens PTd and FHA are formulated based on total protein content (µg/mL). For FHA and PTd, there was no significant difference in the measured antigen concentration between batch 1 and batch 2 of the DTaP vaccine.

In conclusion, targeted mass spectrometry was successfully applied to quantify the antigens present in multivalent vaccines containing aluminum hydroxide adjuvant. The measured FHA concentration differed, depending on the internal standard peptide used. By using the average concentration, it was possible to compare FHA concentrations between both DTaP vaccine batches.

## 4. Discussion

In this study, a mass spectrometry method was developed using antigen-specific signature peptides and standards to quantify the DTd, TTd, PTd and FHA antigens in an aluminumhydroxide-adjuvanted DTaP vaccine product. One or two different signature peptides were selected for quantification of each antigen. A linear relationship was found for the response of the signature peptides versus the antigen concentration. However, the linear concentration range was found to depend on the specific antigen targeted.

A DTaP vaccine containing aluminum hydroxide adjuvant was analyzed for antigen content. Quantification results of the PTd and FHA antigens were consistent with the concentrations supplied by the manufacturer.

During manufacturing, the amounts of DTd and TTd added to the vaccine are based on their flocculation units per ml (Lf/mL), a value derived from an immunological assay measuring the antigenicity of the respective toxoid [26]. The Lf value is not indicative for the amount of toxoid added to the vaccine. The DTd concentration revealed a conversion factor of approximately 2 from Lf to µg, and the TTd concentration gave a conversion factor of about 4 from Lf to µg. This conversion factor could depend on several factors and may be antigen- and/or product-specific. In order to assess the suitability of the developed method for batch-to-batch comparison assessment, two batches of the same DTaP-Hib vaccine were analyzed. Significantly different concentrations for DTd and for TTd between both batches of the DTaP vaccine were measured. The cause of the apparent difference could not be definitively assigned, but may be related to the use of the flocculation assay readout to control the formulation and final vaccine content of these antigens [26].

DTaP vaccines from other manufacturers might contain low amounts of Tween-80 that result in interference during separation on an LC-system (results not shown). Products, such as DetergentOUT™ spin columns, can be used to remove Tween-80 from the vaccine product. Other approaches to remove Tween-80 from vaccine products can be filter-aided sample preparation (FASP) [27] or SCX-fractionation. Although these cleanup methods can be readily implemented in the LC-MS sample preparation workflow, the effect on performance parameters of this assay has not been evaluated.

The quantification of the same target protein was sometimes dependent on the signature peptide used, in particular, for FHA. The cleavage efficiency of the antigens to generate the signature peptides was monitored and was judged to be complete after each digestion, based on the lack of length variants of the signature peptides. The digestion efficiency of individual antigens was determined qualitatively by visualization on SDS-PAGE gels, which showed that all antigens were completely digested, except for FHA, where the antigen degradation was partial. This could explain the difference in FHA concentrations, as obtained with the two different signature peptides, although both peptides are close to each other in the antigen sequence. Digestion of individual antigens in multivalent, adjuvanted vaccines cannot be reliably monitored by SDS-PAGE and the effects of the adjuvant and/or the other antigens on FHA digestion efficiency could explain the observed differences in antigen concentrations between the two signature peptides. The three-dimensional structure of FHA is unknown in the region where the signature peptides are located, making it hard to predict if these peptides can be recovered easily following proteolysis. In light of the challenges with quantification of FHA by LC-MS, it would be interesting to perform quantitative analyses using alternative methods (e.g., ELISA or Luminex) to assess the accuracy of the FHA quantitation using the different peptide standards.

In conclusion, we demonstrated that individual antigens in a multivalent, adjuvanted DTaP vaccine can be identified and specifically quantitated through LC-MS. This approach does not provide direct information on vaccine potency or biological activity but can be used to ultimately demonstrate the consistency of DTaP vaccine batches, as well as individual antigen batches [2]. As such, LC-MS quantitation is a powerful tool for demonstrating vaccine product consistency, and as part of the consistency approach, with the overall objective of reducing the reliance on animal-based testing for vaccine control and release [28]. Further optimization of sample preparation and LC-MS workflows for more reliable quantitation of individual antigens, as well as applicability to other vaccine products containing different adjuvants, such as aluminum phosphate, would enhance the power of this approach. Mass spectrometry is a suitable method to quantify the antigens in a DTaP vaccine adjuvanted with aluminum hydroxide.

## Figures and Tables

**Table 1:** Amino acid sequences and masses of the selected signature peptides used in this study.

SP Number	Peptide Code	Peptide ^a^	Antigen	Antigen Molecular Weight (Average, kDa)	Antigen Accession Number	MH+ Full Length Internal Standard Peptide	MH+ Labeled Signature Peptide Determinant	MH+ Unlabeled Signature Peptide
1	Pep_DTd_01	**DSII[^13^C_6_,^15^N_4_-R]TGFQGESGH**DIKIT	DTd	61.6	P00588	2084.1	1513.7	1503.7
2	Pep_TTd_01	**DLYEKT[^13^C_6_,^15^N_1_-L]N**DYKAI	TTd	150.6	P04958	1592.8	1002.5	995.5
3	Pep_TTd_02	**DTEGFNIES[^13^C_6_,^15^N_2_-K]**DLKSE	TTd	150.6	P04958	1719.8	1147.5	1139.5
4	Pep_FHA_01	**DAG[^13^C_6_,^15^N_1_-L]AGPSAVAAPAVGAA**DVGVE	FHA	243.7	A0A171K3W4	1972.0	1472.8	1465.8
5	Pep_FHA_02	ALR**DVG[^13^C_6_,^15^N_1_-L]EKRL**	FHA	243.7	A0A171K3W4	1276.8	936.5	929.5
9	Pep_PTd_01	**DGTPGGA[^13^C_9_,^15^N_1_-F]**DLKTT	PTd	127.1	P04977 (S1), P04978 (S2), P04979 (S3), P0A3R5 (S4), P04981 (S5)	1289.6	731.3	721.3
10	Pep_PTd_02	**DSP[^13^C_9_,^15^N_1_-Y]PGTPG**DLLEL	PTd	127.1	P04977 (S1), P04978 (S2), P04979 (S3), P0A3R5 (S4), P04981 (S5)	1483.7	900.4	890.4

^a^ Sequence of the stable isotopically labeled synthetic internal standard peptide, with the signature peptide determinant depicted in bold.

**Table 2:** Vaccine products used for antigen quantification, including composition, as provided by the manufacturer.

ID	Vaccine Composition	Experimental Vaccine Composition
Batch 1 vaccine A	50 µg/mL FHA	Full vaccine sample
	50 µg/mL PTd	
	20 Lf/mL TTd	
	60 Lf/mL DTd	
	1.2 mg/mL Al	
Batch 1 vaccine B	50 µg/mL PTd	Control sample
	20 Lf/mL TTd	
	60 Lf/mL DTd	
	1.2 mg/mL Al	
Batch 1 vaccine C	50 µg/mL FHA	Control sample
	20 Lf/mL TTd	
	60 Lf/mL DTd	
	1.2 mg/mL Al	
Batch 1 vaccine D	50 µg/mL FHA	Control sample
	50 µg/mL PTd	
	60 Lf/mL DTd	
	1.2 mg/mL Al	
Batch 1 vaccine E	50 µg/mL FHA	Control sample
	50 µg/mL PTd	
	20 Lf/mL TTd	
	1.2 mg/mL Al	

**Table 3:** Antigen recovery vaccine A batch 1 and batch 2.

Antigen	Average Antigen Concentration Batch 1 (µg/mL) ^1^	Average Antigen Concentration Batch 2 (µg/mL) ^1^	*t*-Test Batch 1 Versus Batch 2
DTd	94 ± 6 (*n* = 36)	137 ± 13 (*n* = 12)	*
TTd	81 ± 4 (*n* = 72)	90 ± 14 (*n* = 24)	*
FHA	68 ± 24 (*n* = 67)	58 ± 16 (*n* = 22)	ns
PTd	35 ± 5 (*n* = 69)	40 ± 14 (*n* = 24)	ns

ns = not significantly different, *p*-value > 0.05. * *p*-value < 0.0005. ^1^ average concentration using all signature peptides.

## Data Availability

The data presented in this study are available on request from the corresponding author. The data are not publicly available due to privacy and institutional policy.

## Supplementary Materials

The following supporting information can be downloaded at: https://www.mdpi.com/article/10.3390/vaccines10071078/s1, Table S1: Sequences of candidate signature peptides for antigen quantification. Figure S1. SDS-PAGE results of digestion for DTd, TTd, FHA and PTd using different enzyme-to-substrate ratios. Figure S2. Quantification of antigens in a second batch of DTaP vaccine.
