# Supplementary material for: Mass Spectrometry-Based Quantification of the Antigens in Aluminum Hydroxide-Adjuvanted Diphtheria-Tetanus-Acellular-Pertussis Combination Vaccines
Source: Vaccines (Basel). 2022 Jul 5;10(7):1078. doi: 10.3390/vaccines10071078 (PMC9323524; doi:10.3390/vaccines10071078)
Supplement: Supplementary file 1 [file vaccines-10-01078-s001.zip › vaccines-1773790-supplementary.pdf]

Supplementary Data

Table S1. Sequences of candidate signature peptides for antigen quantification.

**DTd**				
	**Sequence**	**Position in antigen**	**MH^+ (monoisotopic)**	**Antigen intensity with 1:20 w/w Asp-N ratio**
Pep_DTd_01	DSIIRTGFQGESGH	404-417	1503.7187	1.22E8
Pep_DTd_02	DSIGVLGYQKTV	539-550	1279.6892	7.58E7
Pep_DTd_03	DAAGYSV[2]	62-68	682.3042	NF
Pep_DTd_04	DSETA[1]	291-295	522.2042	6.27E5
Pep_DTd_05	DHTKVNSKLSLFFEIKS[2]	520-536	1993.0753	NF
**TTd**				
Pep_TTd_01	DLYEKTLN	290-297	995.5044	3.64E7
Pep_TTd_02	DTEGFNIESK	396-405	1139.5215	5.81E7
Pep_TTd_03	DPNYLRT	74-80	878.4367	1.02E7
Pep_TTd_04	DTEYYLIPVASSSK	1125-1138	1572.7792	1.90E7
Pep_TTd_05	DPVKIPNLL	381-389	1008.6088	4.68E6
Pep_TTd_06	DVQLKNIT	1139-1146	930.5255	2.85E7
**FHA**				
Pep_FHA_01	DAGLAGPSAVAAPAVGAA	2305-2322	1465.7645	3.92E7
Pep_FHA_02	DVGLEKRL	2214-2221	929.5415	9.61E7
Pep_FHA_03	DATLTLAQGTQR	1661-1672	1274.6699	2.69E7
Pep_FHA_04	DLTAEQSLIEVGK	1855-1867	1402.7424	5.80E7
Pep_FHA_05	DLRTVYAKQA	2076-2085	1164.6372	4.43E7
**PTd**				
Pep_PTd_01	DGTPGGAF	309-316	721.3151	5.84E7
Pep_PTd_02	DSPYPGTPG	772-780	890.3890	5.70E7
Pep_PTd_03	DNNFYGAASSYFEYV	68-82	1746.7282	7.92E6
Pep_PTd_04	DGTYLGGEYGGVIK	295-308	1428.7005	1.98E7
Pep_PTd_05	DAGHEH[1,2]	740-745	665.2638	NF

[1]Not antigen-specific

[2]peptides not suitable as target peptide based on intensity

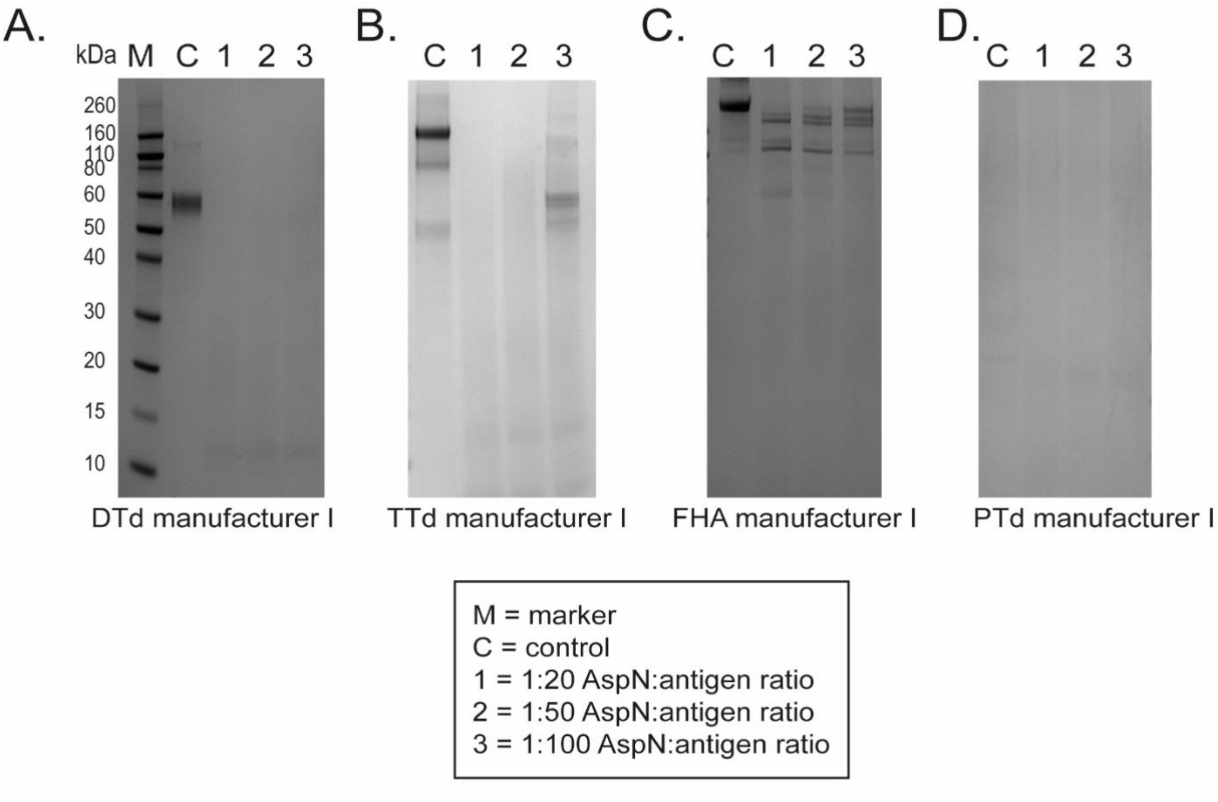

Figure S1. SDS-PAGE results digestion of DTd, TTd, FHA and PTd using different enzyme-to-substrate ratios. Non-adjuvanted samples of individual DTd (A.), TTd (B.), FHA (C.) and PTd (D.) antigens were analyzed.

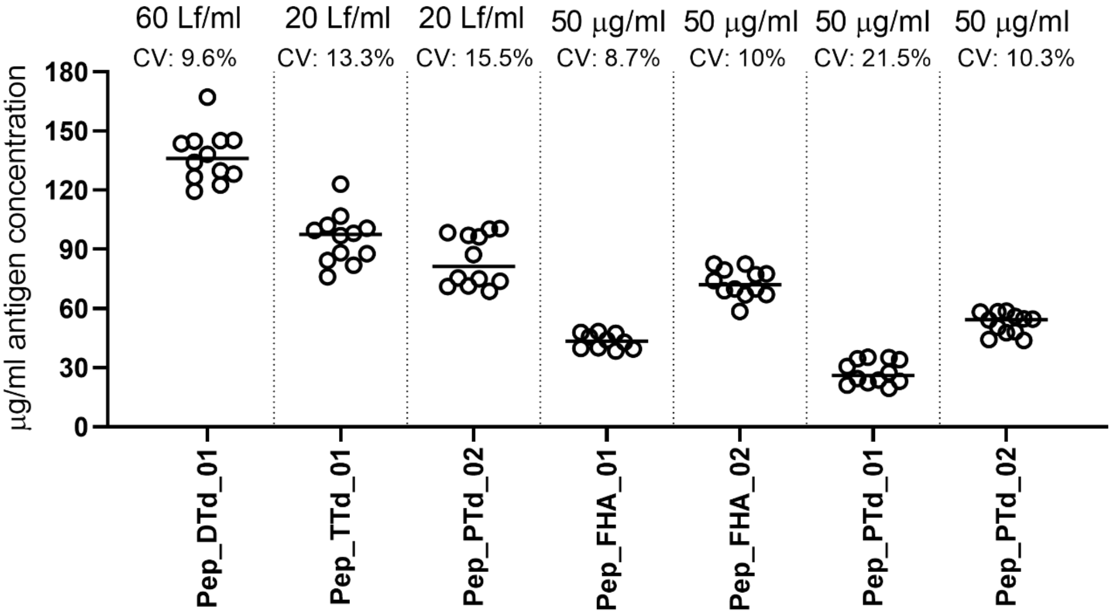

Figure S2. Quantification of antigens in a second batch of DTaP vaccine. The used signature peptides are shown on the X-axis and the antigen concentrations are plotted on the Y-axis. To determine the amount of antigen present in vaccine A batch 2, 100 pmol of each internal standard peptide was added to 100 µl vaccine and digested with 0.33 µg Asp-N (n=12 per signature peptide, except for peptide 4, n=10).
